# Neck circumference to inter-incisor gap ratio: a new predictor of difficult laryngoscopy in cervical spondylosis patients

**DOI:** 10.1186/s12871-017-0346-y

**Published:** 2017-04-04

**Authors:** Yong-zheng Han, Yang Tian, Mao Xu, Cheng Ni, Min Li, Jun Wang, Xiang-yang Guo

**Affiliations:** Department of Anesthesiology, Peking University Third Hospital, Beijing, 100191 China

**Keywords:** Difficult laryngoscopy, Intubation, Cervical spondylosis

## Abstract

**Background:**

Preoperative airway assessment help anticipate a difficult airway. We hypothesized that a close association existed between difficult laryngoscopy and the neck circumference/inter-incisor gap ratio (RNIIG). Our aim was to determine its utility in predicting difficult laryngoscopy in cervical spondylosis patients.

**Methods:**

Two hundred thirteen consecutive patients, aged 20–70 years, scheduled to undergo cervical spine surgery under general anesthesia, were recruited. Preoperative assessments included inter-incisor gap (IIG), thyromental distance (TMD), neck circumference (NC), NC/IIG ratio (RNIIG), NC/TMD ratio (RNTMD) and modified Mallampati test (MMT). Cormack–Lehane scales were assessed during intubation. The anesthesiologist was blinded to the airway assessments. RNIIG’s ability to predict difficult laryngoscopy was compared with that of established predictors.

**Results:**

Difficult laryngoscopy incidence was 16.4%. Univariate analysis showed that male gender, increased age, weight, NC, RNIIG and RNTMD, decreased IIG and TMD, and MMT 3 and 4 were associated with difficult laryngoscopy. Binary multivariate logistic regression analyses identified only one factor that was independently associated with difficult laryngoscopy: RNIIG. The odds ratio and 95% confidence interval (95% CI) were 1.932 (1.504–2.482). RNIIG (≥9.5) exhibited the largest area under the curve (0.80; 95% CI 0.73–0.86) and the highest sensitivity (88.6%; 95% CI 78.1–99.1) and negative predictive value (96.6%; 95% CI 94.0–99.2), confirming its better predictive ability.

**Conclusions:**

RNIIG is a new and simple predictor with a higher level of efficacy, and could help anesthetists plan for difficult laryngoscopy management in cervical spondylosis patients.

**Trial registration:**

ChiCTR-OON-16008320 (April 19th, 2016).

## Background

Airway management is crucial in clinical anesthesia. The incidence of difficult laryngoscopy and intubation in various settings has ranged widely, from 1 to 15% [1–3]. For patients undergoing surgery for cervical spondylosis, they had impaired cervical mobility. Considering the fact that the best laryngoscopy view is achieved when the oral, pharyngeal, and laryngeal axes are most closely matched, it was difficult for an anesthesiologist to expose the glottis in cervical spondylosis patients. Therefore, compared with other patients, cervical spondylosis patients have a higher incidence of difficult laryngoscopy which causes a large proportion of unexpected difficult airways and significantly increases the morbidity and mortality rates [4]. Therefore, identifying a more reliable optimal predictor of difficult laryngoscopy in cervical spondylosis patients undergoing general anesthesia is desirable.

Suggested predictors for difficult laryngoscopy include increased neck circumference, high score of Mallampati test (MMT), small inter-incisor gap (IIG) and thyromental distance (TMD) [5]. All of these techniques are relatively quick bedside tests that are easy to perform with no special equipment. However, none of them alone has high diagnostic accuracy, particularly in cervical spondylosis patients.

Many studies found that modifications to bedside tests have shown improved predictive values. An example includes the ratio of neck circumference to TMD (RNTMD). When comparing independent predictors of difficult tracheal intubation in obese patients including the Mallampati test and the Wilson score, the RNTMD had the best predictive outcome [6]. IIG is a major single predictor for difficult airways. It reflects craniocervical extension, which is restricted in cervical spondylosis patients [7]. In our former study of cervical spondylosis patients [8], we found that IIG could predict difficult airway better than TMD. Therefore, our hypothesis was that the NC/IIG ratio (RNIIG) would be more useful than the previously reported indices for identifying difficult intubation in cervical spondylosis patients.

## Methods

After institutional ethics committee approval (IRB00006761-2015021, Medical Ethics Committee of Peking University Third Hospital), informed written consent was obtained from all patients. We recruited 213 patients (20–70 years of age) undergoing cervical spine surgery with general anesthesia between April 2016 and October 2016. Patients met the following criteria: mentally competent adult and American Society of Anesthesiologists (ASA) score I–III; requirement for preoperative neck immobilization with a hard plastic collar; elective cervical spine surgery for severe cervical spondylosis. We excluded patients with pregnancy or oropharyngeal mass.

Clinical tests for the preoperative airway assessment and demographic information—gender, age, height, weight, body mass index (BMI)—were recorded 1 day before surgery by one anesthesiologist who was not involved in the anesthesia induction (to avoid inter-observer variability).

The difficulty of laryngoscopy was assessed with the Cormack–Lehane (C–L) scale, the results of which were recorded by another senior anesthetist. The C–L scale is graded in Table 1. Those with class III–IV and class I–II were defined as the difficult and easy laryngoscopy groups, respectively [9].

**Table 1:** The Cormack–Lehane (C–L) scale

Classification	Description
Class I	vocal cords were completely visible
Class II	only the arytenoids were visible
Class III	only the epiglottis was visible
Class IV	the epiglottis was not visible

The IIG (distance between the upper and lower incisors at the midline) was measured by asking each patient to open the mouth to the maximum extent. The TMD (distance from the thyroid notch to the mentum) was measured with the neck extended. Neck circumference at the level of the cricoid cartilage was measured. The RNIIG and RNTMD were then calculated. The oropharyngeal view was assessed using a MMT classification. The patients were asked to sit, open their mouths fully, and protrude the tongue without phonation [10]. The cases were rated in Table 2.

**Table 2:** The modified Mallampati test (MMT)

Classification	Description
Class I	the soft palate, fauces, uvula, and pillars were visible
Class II	the soft palate, fauces, and uvula were visible
Class III	the soft palate and the base of the uvula were visible
Class IV	the soft palate was not visible

No premedication was allowed. Preoperative routine monitoring included noninvasive blood pressure, heart rate, pulse oximetry, and electrocardiography. Anesthesia was induced with sufentanil (0.3 μg/kg) and propofol (2 mg/kg). Patients were manually ventilated following loss of consciousness. Neuromuscular blockade was achieved by injection of rocuronium (0.6 mg/kg). The C–L grade was determined during direct laryngoscopy, in all patients in the sniff position, by the same senior anesthesiologist not involved in the preoperative assessment. After that, tracheal intubation was then performed with the direct laryngoscope or alternative devices by the same anesthesiologist. In patients with difficult airway, intubation was completed following ASA guidelines [11].

Estimating a 10% incidence of difficult laryngoscopy [12], a sample size of 189 patients was calculated to have at least 90% power to detect a difference in predictors between the difficult and easy laryngoscopy groups estimated with PASS software (version 8.03; NCSS LLC, Kaysville, UT, USA). In consideration of the dropout, 213 patients were recruited in our study finally. SPSS software (version 21.0; IBM Corp, Armonk, NY, USA) was used for statistical analysis. Categorical variables were analyzed using the *χ* ^2^ test, and continuous variables were analyzed by an independent samples *t*-test. Binary multivariate logistic regression analyses were performed to identify multivariate predictors for difficult airways. A receiver operating characteristic (ROC) curve was used to describe the discrimination abilities of the predictive indicators. The area under the curve (AUC) was used as the quantitative index to describe the ROC curve. The 95% CI was calculated, and *P* < 0.05 was considered to indicate statistical significance.

## Results

A total of 213 patients comprising 141 men (66.2%) and 72 women (33.8%) were included in the study. The overall incidence of difficult laryngoscopy was 16.4% (35/213). Univariate analysis demonstrated several risk factors that were associated with difficult laryngoscopy: male gender, increased age, weight, NC, RNIIG and RNTMD, decreased IIG and TMD, and MMT 3 and 4 (*P* < 0.05). The demographic variables and clinical tests for the study population are presented in Table 3.

**Table 3:** Demographics and clinical predictors of the easy and difficult laryngoscopy groups

Characteristic	Easy laryngoscopy group (*n* = 178)	Difficult laryngoscopy group (*n* = 35)	*P* values
Age (years)	52 ± 11	57 ± 10	0.031
Gender (male/female)	112(63)/ 66(37)	29(83)/ 6(17)	0.023
Height (cm)	167.0 ± 8.0	169.5 ± 7.5	0.082
Weight (kg)	69.9 ± 11.8	74.1 ± 9.4	0.045
BMI (kg/m^2^)	25.0 ± 3.5	25.7 ± 2.2	0.115
IIG (cm)	4.3 ± 0.6	3.9 ± 0.5	<0.001
TMD (cm)	9.0 ± 1.2	8.4 ± 1.5	0.012
NC (cm)	39.5 ± 4.1	41.9 ± 3.1	0.001
RNIIG	9.2 ± 1.5	11.0 ± 1.6	<0.001
RNTMD	4.4 ± 0.7	5.1 ± 0.9	<0.001
MMT (class I II/class III IV)	127(71)/51(29)	15(43)/ 20(57)	0.001

Data are presented as mean ± standard deviation or number (proportion, %)

*Abbreviations*: *BMI* body mass index, *IIG* inter-incisor gap, *TMD* thyromental distance, *NC* neck circumference, *RNIIG* the neck circumference/inter-incisor gap ratio, *RNTMD* the neck circumference/thyromental distance ratio, *MMT* modified Mallampati test

Binary multivariate logistic regression (forward-Wald) analyses identified only one independent risk factor from the general condition and clinical tests that correlated best as a predictor of a difficult airway: RNIIG. The odds ratio (OR) and 95% CI were 1.932 (1.504–2.482) (Table 4).

**Table 4:** Predictors for difficult laryngoscopy identified by binary multivariate logistic regression (forward-Wald) model

Variable	β	SE	*P*-value	OR	95% CI
RNIIG	0.658	0.128	<0.001	1.932	1.504–2.482
Constant	−8.236	1.352	<0.001		

*Abbreviations*: *RNIIG* the neck circumference/inter-incisor gap ratio, *SE* standard error, *OR* Odds ratio, *95%CI* 95% confidence interval

The AUC and standard error calculated for those clinical tests are shown in Table 5. We used the ROC curve and AUC to identify the predictive abilities of the clinical predictors. The AUC for RNIIG was higher than those obtained for RNTMD, IIG, NC, MMT, and TMD.

**Table 5:** Predictive values of tests for predicting difficult laryngoscopy

Indicators	AUC	95% CI	SE
RNIIG	0.80	0.73–0.86	0.03
RNTMD	0.74	0.65–0.84	0.05
IIG	0.71	0.63–0.79	0.04
NC	0.69	0.61–0.77	0.04
MMT	0.67	0.58–0.77	0.05
TMD	0.66	0.55–0.76	0.05

*Abbreviations*: *AUC* area under the curve, *95%CI* 95% confidence interval, *SE* standard error, *RNIIG* the neck circumference/inter-incisor gap ratio, *RNTMD* the neck circumference/thyromental distance ratio, *IIG* inter-incisor gap, *NC* neck circumference, *MMT* modified Mallampati test, *TMD* thyromental distance

True-positive (TP), true-negative (TN), false-positive (FP), and false-negative (FN) results, together with the sensitivity, specificity, positive predictive value (PPV), and negative predictive value (NPV) calculated for those clinical tests are shown in Table 6. Compared with the other tests, RNIIG (≥9.5) had the highest sensitivity (88.6%; 95% CI 78.1–99.1) and negative predictive value (96.6%; 95% CI 94.0–99.2), confirming its better predictive ability.

**Table 6:** Evaluation of diagnostic tests for difficult airways

Indicators	TP	TN	FP	FN	Sensitivity (95% CI)	Specificity (95% CI)	PPV (95% CI)	NPV (95% CI)
RNIIG (≥9.5)	31	112	66	4	88.6% (78.1–99.1%)	62.9% (55.8–70.0%)	32.0% (16.1–47.9%)	96.6% (94.0–99.2%)
RNTMD (>4.6)	28	112	66	7	80.0% (66.7–93.3%)	62.9% (55.8–70.0%)	29.8% (20.6–39.0%)	94.1% (89.9–98.3%)
NC (≥41 cm)	23	112	66	12	65.7% (50.0–81.4%)	62.9% (55.8–70.0%)	25.8% (16.7–34.9%)	90.3% (85.1–95.5%)
MMT (III–IV)	20	127	51	15	57.1% (40.7–73.5%)	71.3% (64.7–77.9%)	28.2% (12.8–43.6%)	89.4% (84.9–93.9%)
IIG (≤3.5 cm)	10	155	23	25	28.6% (13.6–43.6%)	87.1% (82.2–92.0%)	30.3% (14.6–46.0%)	86.1% (81.0–91.2%)
TMD (≤7.0 cm)	7	166	12	28	20.0% (6.7–33.3%)	93.3% (89.6–97.0%)	36.8% (20.3–53.3%)	85.6% (80.5–90.7%)

*Abbreviations*: *TP* true-positive, *TN* true-negative, *FP* false-positive, *FN* false-negative, *PPV* positive predictive value, *NPV* negative predictive value, *95%CI* 95% confidence interval, *RNIIG* the neck circumference/inter-incisor gap ratio, *RNTMD* the neck circumference/thyromental distance ratio, *NC* neck circumference, *MMT* modified Mallampati test, *IIG* inter-incisor gap, *TMD* thyromental distance

## Discussion

“An anaesthetist must assess the patient before anaesthesia and devise an appropriate plan of anaesthetic management” – The Good Anaesthetist, Royal College of Anaesthetists 2010 [13]. Airway assessment consists of taking a medical history, performing a physical examination, reviewing the clinical records and carring out additional tests. Successful identification of physical features that are suggestive of a difficult airway should direct planning toward safe airway management. Accordingly, many national airway guidelines emphasize the importance of a thorough and skilled assessment of all patients undergoing anesthesia [14, 15].

Certain single predictors such as Mallampati test and thyromental distance, performed at the bedside in seconds, can be used for routine screening in the general population, but are inadequate in patients undergoing surgery for cervical spondylosis. In these patients, there are a high incidence of difficult airway and few reliable tests to evaluate the difficult airway. In this study, the incidence of difficult laryngoscopy was 16.4%, which is higher than what has been reported in previous studies [2]. Bedside airway tests have been criticized for their poor predictive capability, the poor predictive capability relates to the high incidence of unanticipated difficulty airway. In this study, we found that RNIIG was a more accurate method for predicting difficult laryngoscopy in these patients.

Our data demonstrated that male gender, increasing weight and age were associated with difficult laryngoscopy. A significantly greater proportion of difficult tracheal intubations had been found in men and obesity patients [16, 17], which were attributed to differences in neck fat deposition between the genders [18]. An association between difficult laryngoscopy and older patients has also been reported [19]. Osteoarthritic changes, associated with decreased thyromental distance, cervical spine movement, interincisor distance, and grade of dentition, may be responsible for age-related increases in difficult laryngoscopy. Although patients in the difficult laryngoscopy group were heavier than those in the easy laryngoscopy group, BMI didn’t show statistically significant risk for difficult laryngoscopy. The results were in accordance with the study reported by Prakash et al. [20]. In a cohort study from the Danish Anesthesia Database of 91,332 consecutive patients, by examining the relative impact of weight and height, only weight was found as an independent risk factor for difficult tracheal intubation [21].

The Mallampati test [22] and thyromental distance [23] are all impaired by cervical spine limitation in cervical spondylosis patients which suggests the importance of adequate neck movement when trying to predict difficult airway. MMT could reflect oropharyngeal cavity volume, but it could not assess laryngeal condition. As for TMD, short TMD is a surrogate for inadequate head extension, rather than small submandibular space, when indicating possible difficult direct laryngoscopy. Both of MMT and TMD had lower AUC in our study. The results of the current study support those previous studies regarding poor sensitivity values for the MMT and TMD tests [24, 25].

The IIG has been demonstrated to be one of the most sensitive single predictors of a difficult airway in normal patients [26]. Craniocervical extension occurs during normal mouth opening, and nearly maximum mouth opening was obtained with 26° (95% CI 22–30) of craniocervical extension from the neutral position [7]. Craniocervical extension is an integral part of complete mouth opening in conscious subjects, whereas mouth opening may be restricted in patients with cervical spondylosis. However, the sensitivity of the IIG was not high enough (28.6%), suggesting that it was not reliable as a single predictor.

Ezri et al. reported that the abundance of pre-tracheal soft tissue at the level of the vocal cords was a good measure that fully distinguished an easy laryngoscopy from a difficult one in obese patients [16]. The accumulation of pre-tracheal soft tissue might cause difficult laryngeal exposure. The NC alone might not clearly indicate the amount of soft tissue at various topographical regions within the neck. Therefore, the NC might not be a perfect indicator. Our study also confirmed that NC was a moderate predictor, with 65.7% sensitivity and 0.689 AUC.

Kim et al. [6] found that RNTMD was a better indicator than either the NC or TMD alone. It was more difficult for patients with both a large neck circumference and a short neck to be given intubation than patients with a large neck circumference or a short neck alone. Previous studies have suggested that using a single screening test for difficult intubations only provides limited accuracy [27]. However, combinations of tests may increase the accuracy of diagnosis. The El-Ganzouri or Wilson scores [28, 29] are multivariate risk index systems, but they are time consuming as they contain multiple risk factors. Thus, combining two of the most valuable risk factors may add some incremental diagnostic value while not significantly increasing the burden of testing.

RNIIG is a new predictor of difficult laryngoscopy in cervical spondylosis patients, it contained two indicators: IIG and NC, which represented oropharyngeal cavity volume, craniocervical extension and laryngeal cavity volume. Compared with RNTMD, IIG, NC, MMT and TMD, RNIIG showed a relatively large AUC on the ROC curve which revealed that the RNIIG is highly predictive. The AUC for IIG was higher than TMD, so it might be the reason why RNIIG was better than RNTMD. RNIIG had higher sensitivity than any other test, thus providing fewer false-negative predictions. In other words, RNIIG missed the least number of difficult laryngoscopies. The failure of a test to predict a difficult case (false-negative result) could result in unanticipated difficult airway and life-threatening events. Binary multivariate logistic regression (forward-Wald) analyses identified only one independent risk factor from among the general condition and clinical tests that correlated best as a predictor of a difficult airway: RNIIG. The ORs and 95% CI were 1.932 (1.504–2.482). Wilson model is the most commonly used multivariate clinical model for predicting difficult airway. Naguib et al. [30] reported that the AUC and sensitivity of Wilson model were 0.79 (95% CI, 0.72–0.85) and 40.2% (95% CI, 30.0–50.0%). In our study, the AUC and sensitivity of RNIIG were 0.80 (95% CI, 0.73–0.86) and 88.6% (95% CI, 78.1–99.1%) suggesting that RNIIG showed better performance than Wilson model, and it could be more useful as a simple screening test for cervical spine surgery. In clinical practice, if we find the RNIIG of a cervical spondylosis patient is more than 9.5, it is suggested that we may encounter a difficult laryngoscopy. Therefore, we should follow the practice guidelines for management of the difficult airway recommended by the American Society of Anesthesiologists task force. Be sure that a senior anesthesiologist and visual tools (video laryngoscope, shikani optical stylet, fiberoptic bronchoscope, et al.) are all standby rather than rapid induction of general anesthesia with Macintosh laryngoscope for intubation.

Our study had some limitations. First, it was not completely blinded as the anesthesiologist could recognize the patients’ characteristics in the operating theater. It was thus impossible to maintain complete blindness during this study. Second, determination of the best cutoff point as a difficult laryngoscopy predictor, and its analysis as a measure of prediction, had both been performed on the same population. This might influence the statistical results to a certain extent. Third, we calculated the ideal cutoff values for RNIIG and RNTMD from the data obtained in the study, whereas the cutoff values for the other tests were obtained from the literature. Therefore, the ideal predictor (RNIIG) and its cutoff value should be tested in a future study.

## Conclusions

We found a 16.4% incidence of difficult laryngoscopy in cervical spondylosis patients. RNIIG was the only independent risk factor for difficult laryngoscopy. RNIIG of ≥9.5 yielded moderate to high sensitivity, specificity, and negative predictive value. Thus, we consider a preoperative value of RNIIG ≥9.5 to be a good predictor of difficult laryngoscopy in cervical spondylosis patients.

## Abbreviations

95% CI: 95% confidence interval
AUC: Area under the curve
C–L scale: Cormack–Lehane scale
IIG: Inter-incisor gap
MMT: Modified Mallampati test
NC: Neck circumference
RNIIG: NC/IIG ratio
RNIIG: The neck circumference/inter-incisor gap ratio
RNTMD: NC/TMD ratio
ROC curve: Receiver operating characteristic curve
TMD: Thyromental distance
